# Comprehensive characterization of pathological stage‐related genes of papillary thyroid cancer along with survival prediction

**DOI:** 10.1111/jcmm.16799

**Published:** 2021-08-02

**Authors:** Lei Xu, Feng Liu, Haiyan Li, Menglong Li, Yongmei Xie, Zhihui Li, Yanzhi Guo

**Affiliations:** ^1^ College of Chemistry Sichuan University Chengdu China; ^2^ Department of Thyroid Surgery West China Hospital of Sichuan University Chengdu China; ^3^ Laboratory of Thyroid and Parathyroid Disease Frontiers Science Center for Disease‐related Molecular Network West China Hospital of Sichuan University Chengdu China

**Keywords:** hub gene, papillary thyroid cancer, prognostic risk model, tumour stage

## Abstract

It is crucial to understand the differences across papillary thyroid cancer (PTC) stages, so as to provide a basis for individualized treatments. Here, comprehensive function characterization of PTC stage‐related genes was performed and a new prognostic signature was developed for advanced patients. Two gene modules were confirmed to be closely associated with PTC stages and further six hub genes were identified that yield excellent diagnostic efficiency between tumour and normal tissues. Genetic alteration analysis indicates that they are much conservative since mutations in the DNA of them rarely occur, but changes of DNA methylation on these six genes show that 12 DNA methylation sites are significantly associated with their corresponding genes' expression. Validation data set testing also suggests that these six stage‐related hub genes would be probably potential biomarkers for marking four stages. Subsequently, a 21‐mRNA‐based prognostic risk model was constructed for PTC stage III/IV patients and it could effectively predict the survival of patients with strong prognostic ability. Functional analysis shows that differential expression genes between high‐ and low‐risk patients would promote the progress of PTC to some extent. Moreover, tumour microenvironment (TME) of high‐risk patients may be more conducive to tumour growth by ESTIMATE analysis.

## 1 INTRODUCTION

As the most common type of endocrine tumour, thyroid cancer is one of malignant tumours whose incidences are rapidly increasing.^1^, ^2^, ^3^ It includes four main subtypes: papillary thyroid carcinoma (PTC), follicular thyroid carcinoma (FTC), medullary thyroid carcinoma (MTC) and anaplastic thyroid carcinoma (ATC).^4^ Of them, PTC is the most major type accounting for more than 80% of all cases.^2^ PTC tumours can be clinically divided into four pathological stages (I, II, III and IV). Generally, prognosis of patients with PTC is excellent with 5‐year survival rate over 97%.^5^ However, in stage IV, the 5‐year survival rate of PTC sharply reduces to 51%.^6^, ^7^

Thus, it is of great significance to identify key genes related to PTC stages and understand their biological functions. Meanwhile, the survival rate of patients in advanced stage is significantly lower than that of patients in early stage. Therefore, it is more important to conduct risk stratification analysis for advanced patients to find prognostic factors related to their survival prognosis.

The weighted gene co‐expression network analysis (WGCNA) is deemed as an efficient network‐based approach, which can investigate the signature of gene networks in the pathogenesis of complicated diseases at system level.^8^ It is an algorithm that constructs scale‐free gene co‐expression networks based on the expression of genes, which can not only classify different gene modules, but also figure out the relationships between clinical features and gene modules,^9^ so this method provides an effective way to explore the interaction mechanism of clinical traits‐related genes of diseases and identify potential biomarkers.^7^, ^10^, ^11^, ^12^

Until now, no comprehensive investigation on PTC stage‐related genes has been reported and the regulation characteristics of them are not well revealed. Here, this study first gives systematic functional analysis on them. First, 1243 common differentially expressed genes (DEGs) were screened out by comparing stage I, II, III and IV PTC samples with adjacent non‐tumour tissue samples. Then, WGCNA was employed to study the co‐expression network of DEGs and two gene modules were proved to be associated with tumour stages. The Gene Ontologies (GO) and Kyoto Encyclopedia of Genes and Genomes (KEGG) pathway enrichment analysis show that genes in both these two modules are mainly enriched in cancer‐related pathways, so 6 tumour stage‐related hub genes were identified from the two gene modules, including RPS6KA6, SORBS2, EPHB3, QSOX1, S100A6 and UNC5CL. To validate six hub genes, their expression levels at different stages and the receiver operating characteristic (ROC) diagnostic analysis were, respectively, performed based on validation data sets. Meanwhile, DNA mutation and methylation analyses of the six hub genes were also systemically implemented.

Besides, we established a 21‐mRNA‐based prognostic risk model for PTC patients with stage III and IV using a least absolute shrinkage and selection operator (LASSO) Cox method. Kaplan‐Meier analysis, ROC analysis, Cox regression analysis and stratified analysis were employed to assess and validate the prediction performance of the risk model on the overall survival (OS) of advanced patients. Finally, we used KEGG pathway analysis and ESTIMATE analysis to explore the changes of biological pathways and TME between high‐risk and low‐risk patients.

## 2 MATERIALS AND METHODS

### 2.1 Samples and preprocessing

The transcriptome data (level 3, HTSeq‐counts) and clinical information of PTC patients were downloaded from TCGA data portal (https://portal.gdc.cancer.gov/). Then, the analysis samples were cleaned by removing those with other tumours and lacking of clinical and tumour stage annotation, so 470 PTC patients were remained, including 270 stage I, 50 stage II, 100 stage III and 50 stage IV samples. Of them, 56 samples contain both tumour and adjacent non‐tumour tissue samples.

According to the annotation information of gene type from GENCODE Version 29 (https://www.gencodegenes.org/), the gene expression data of 19645 mRNAs were extracted. Then, genes with no or low expression in more than a quarter of the samples (read count <10) were discarded, so 14647 mRNAs were remained. Next, they were normalized by Trimmed Mean of M values (TMM).^13^As validation data, two microarray data sets GSE29265 and GSE3678 were downloaded from GEO database, including 10 and 7 pairs between tumour and normal tissues, respectively.

### 2.2 Differential gene expression analysis

DEGs were detected using edgeR package in R software (http://bioconductor.org/packages/edgeR/).^14^ DEGs of stage I, II, III and IV PTC samples compared with adjacent non‐tumour tissue samples were, respectively, screened out, according to the cut‐off criteria of absolute log_2_ (fold change; |log_2_FC|) ≥1 and false discovery rate (FDR) <0.05. Then, common DEGs were achieved by overlapping the four groups' DEGs.

### 2.3 Weighted gene co‐expression networks construction

Weighted gene co‐expression network analysis was performed using ‘WGCNA’ R package.^9^ First, the samples were clustered to delete the outlier samples. Second, a soft‐threshold power β was selected based on the criterion of approximate scale‐free topology using the function pickSoftThreshold. Third, the adjacency was transformed into a topological overlap matrix (TOM) using function TOM similarity. Fourth, according to the TOM‐based dissimilarity measurement, average linkage hierarchical clustering was conducted to produce the common DEGs dendrogram. Consequently, module identification was performed with the function cutreeDynamic (minModuleSize of 30). Finally, to further analyse the module, the dissimilarity of module eigengenes (MEs) was calculated using the function moduleEigengenes. ME is defined as the first principal component of the gene expression matrix of the corresponding module, which can summarize the gene expression profiles from a module. Highly similar modules were identified by clustering analysis and then to be merged together with a height cut‐off of 0.25.

### 2.4 Identification of stage‐related gene modules and hub genes

To identify the stage‐related modules and genes, module–trait relationship analysis was performed to measure the correlation between clinical traits and gene modules. The Pearson correlation coefficient and *p* value were calculated by between ME and clinical trait. The results were presented using heat map. Next, gene significance (GS) was calculated based on the correlation of a gene expression profile with a clinical trait. In general, the higher the absolute GS, the higher the correlation between this module and the clinical trait. For a certain gene, its Module membership (MM) was defined as correlation between its expression profile in all samples and the expression profile of a certain modules (MEs). The greater the MM value of the gene, the more important the gene is in the module. We defined the thresholds for the selection of hub genes as MM >0.8 and GS >0.2. In order to explore the potential biological mechanism of each module, the genes in each module were uploaded into KOBAS (http://kobas.cbi.pku.edu.cn/kobas3),^15^ which is an online tool for gene enrichment analysis. Then, the Gene Ontologies (GO) functional enrichment analysis and Kyoto Encyclopedia of Genes and Genomes (KEGG) pathway enrichment were performed. Corrected *p* value <0.05 was set as the cut‐off criteria.

Gene expression difference analysis was used to validate the practicability of hub gene as biomarkers. On one hand, the expression of hub genes in PTC was studied using Gene Expression Profiling Interactive Analysis (GEPIA)^16^ data sets. On the other hand, hub gene expression levels at different stages were also plotted. In addition, the receiver operating characteristic (ROC) curve was preformed to verify the diagnostic performance of hub genes using ‘pROC’ package (https://cran.r‐project.org/web/packages/pROC/).

### 2.5 Genetic alteration analysis of hub genes

Genetic alterations about hub genes were explored using cBioPortal (http://www.cbioportal.org/).^17^ In addition, the changes of DNA methylation sites on hub genes were studied. First, DNA methylation data of the sites locating in hub genes were extracted; then, with data filtering and difference analysis, different methylation sites (DMSs) were selected according to the threshold that |Δβ| > 0.1 and *p* value <0.05 (Δβ: the difference value between the average β values of tumour and normal tissues); last, the Spearman's rank correlation coefficients between these DMSs and their genes were calculated.

### 2.6 Construction and evaluation of the prognostic model for PTC advanced patients

A total of 148 samples with tumour stage III and stage IV were subjected to prognostic modelling analysis, after removing two with follow‐up time less than 1 month. A two‐step analysis strategy was established for prognostic modelling. First, the common DEGs were selected to analyse their relationship with OS of PTC patients by univariate Cox regression analysis. Those with *p* value <0.05 were extracted. Second, a least absolute shrinkage and selection operator (LASSO) Cox penalized regression model^18^ was preformed to build the classifier using R package ‘glmnet’ (https://cran.r‐project.org/web/packages/glmnet/).^19^In order to optimize the model, 10‐fold cross‐validation was employed. Finally, candidate genes with non‐zero coefficient were filtered to build a prognostic model. The risk score of each PTC patient was calculated by the following formula:

(1) $$RiskScore=\sum_{i=1}^{N}\left(Ei\times Ci\right)$$

where *N* is the number of candidate genes, E*i* is the expression of candidate normalized by TMM, and C*i* is the coefficient of candidate genes in the LASSO Cox regression analysis.

Based on the risk score, the PTC patients were divided into high‐ and low‐risk groups by cut‐off median. A Kaplan‐Meier survival curve was employed for survival analysis, and log‐rank tests were used to compare the differences of OS between two groups. Meanwhile, time‐dependent ROC analysis was used to investigate the prognosis accuracy of the model and area under the ROC curve (AUC) values were also calculated using the ‘timeROC’ package (https://cran.r‐project.org/web/packages/timeROC/).

The stratified analysis was conducted to determine whether the prognostic signature is independent of other clinical factors. KEGG pathway enrichment analysis was conducted on DGEs between high‐ and low‐risk groups to explore potential biological pathway alteration. In addition, the stromal score, immune score and ESTIMATE score for each patient with PTC were computed using ‘estimate’ package (https://bioinformatics.mdanderson.org/estimate/).^20^

## 3 RESULTS

### 3.1 Screening DEGs

**FIGURE 1:**
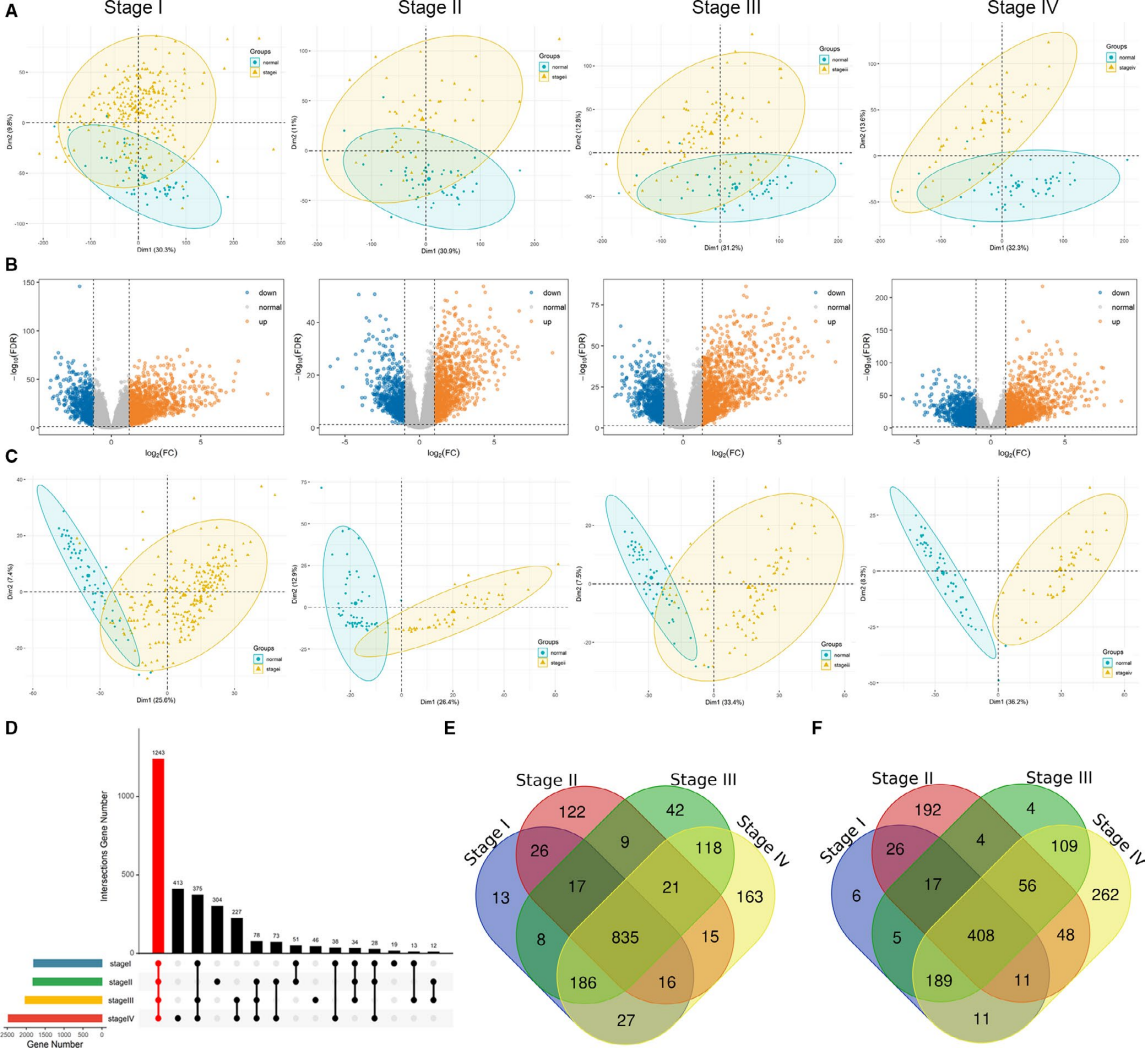
The distributions of differentially expressed genes. (A) PCA for different tumour stages tissues and normal tissues using all gene expression data. (B) Volcano plots of four comparison groups' differentially expressed genes. (C) PCA for different tumour stages tissues and normal tissues using differentially expressed genes data. (D) Upset plot of differentially expressed genes in four comparison groups. (E) Veen plots of up‐regulated genes in four comparison groups. (F) Veen plots of down‐regulated genes in four comparison groups

We first performed principal component analysis (PCA) for different tumour stage tissues and normal tissues using all the filtered and normalized gene expression data. As shown in Figure 1A, normal tissues and tumour tissues at different stages can be separated to a certain extent, but there is still a large proportion of overlaps. Using differential gene expression analysis, we obtained 1801 DEGs (1128 up‐regulated and 673 down‐regulated) between stage I and normal tissues, 1823 DEGs (1061 up‐regulated and 762 down‐regulated) between stage II and normal tissues, 2028 DEGs (1236 up‐regulated and 792 down‐regulated) between stage III and normal tissues, and 2475 DEGs (1381 up‐regulated and 1094 down‐regulated) between stage IV and normal tissues (Figure 1B). Again, these DEGs were used for PCA analysis of PTC tissues at different stages and normal tissues. As shown in Figure 1C, normal tissues were significantly separated from different stages tissues, showing that the DEGs screened here are reliable.

Next, as shown in Figure 1D, 1243 common DEGs were extracted from the four comparison groups. Among these 1243 common DEGs, 835 genes were up‐regulated (Figure 1E) and 408 genes were down‐regulated (Figure 1F). It is obviously to see that either the expression levels of up‐regulated or the down‐regulated DEGs are all consistent in four stages.

### 3.2 Identification of co‐expression gene modules and functional annotation

**FIGURE 2:**
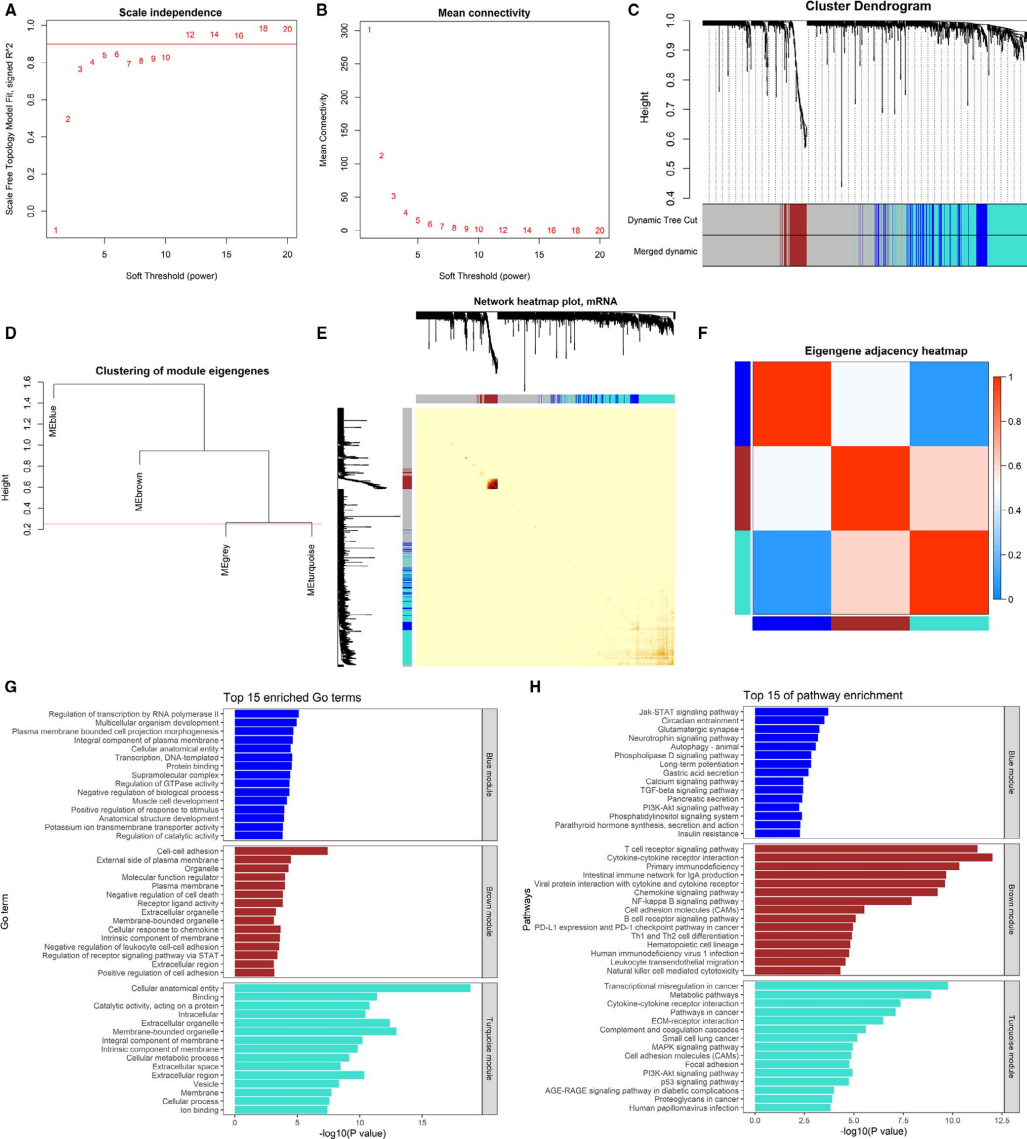
Construction of WGCNA co‐expression modules and functional enrichment analysis of each module. (A‐B) Analysis of network topology for various soft‐thresholding powers. (C) The cluster dendrogram of the common differentially expressed genes in TCGA. Each branch in the figure represents one gene, and every colour below represents one co‐expression module. (D) The cluster dendrogram of module eigengenes. (E) Interaction relationship analysis of co‐expression genes. Different colours of horizontal axis and vertical axis represent different modules. (F) Correlation heatmap of modules' eigengene. (G) The top 15 GO terms of each module. (H) The top 15 KEGG pathway of each module

1243 common DEGs were performed WGCNA analysis. 470 samples of PTC were first clustered to remove obvious outlier samples (Figure S1). To ensure a scale‐free network, the power of β = 12 (*R* ^2^ = 0.952) was chosen for the soft‐threshold parameter (Figure 2A,B). Dynamic hybrid cutting was conducted to construct a hierarchical clustering. The genes with similar expression pattern formed a gene module, so four modules (blue, brown, turquoise and grey) were generated (Figure 2C). Because the similarity between all modules is less than 0.75, there is no module merge (Figure 2D). In addition, the weighted network and the eigengene heatmap were constructed to identify interaction relationships of the four co‐expression modules. Figure 2E,F reveal that each module is independent in the network.

Genes in Grey module were not co‐expressed with genes in any module and they do not co‐express each other, we focused on other three ones. Biological functions of each module were explored by GO and KEGG pathway enrichment analysis. Top 15 GO terms and KEGG pathways for each module are shown in Figure 2G,H. For 82 genes in brown module, the enriched GO terms are ‘Cell‐cell adhesion’, ‘Cellular response to chemokine’ and ‘Positive regulation of cell adhesion’. KGEE pathways are ‘T cell receptor signalling pathway’, ‘Primary immunodeficiency’ and ‘Intestinal immune network for IgA production’. It indicates that these genes are associated with immune reaction. 126 genes in blue module were significantly enriched in ‘Regulation of transcription by RNA polymerase II’ and ‘Multicellular organism development’ with KEGG pathways of ‘Jak‐STAT signalling pathway’ and ‘PI3K‐Akt signalling pathway’, which are common pathways related to cancers. Similar with those in blue module, 397 genes in turquoise module are mainly involved in cancer‐related pathways, including ‘Transcriptional misregulation in cancer’, ‘Pathways in cancer’, ‘Small cell lung cancer’, ‘PI3K‐Akt signalling pathway’, ‘p53 signalling pathway’ and ‘Proteoglycans in cancer’. It indicates that the genes in blue and turquoise modules may have key roles in development and progression of PTC.

### 3.3 Identification of stage‐related modules

**FIGURE 3:**
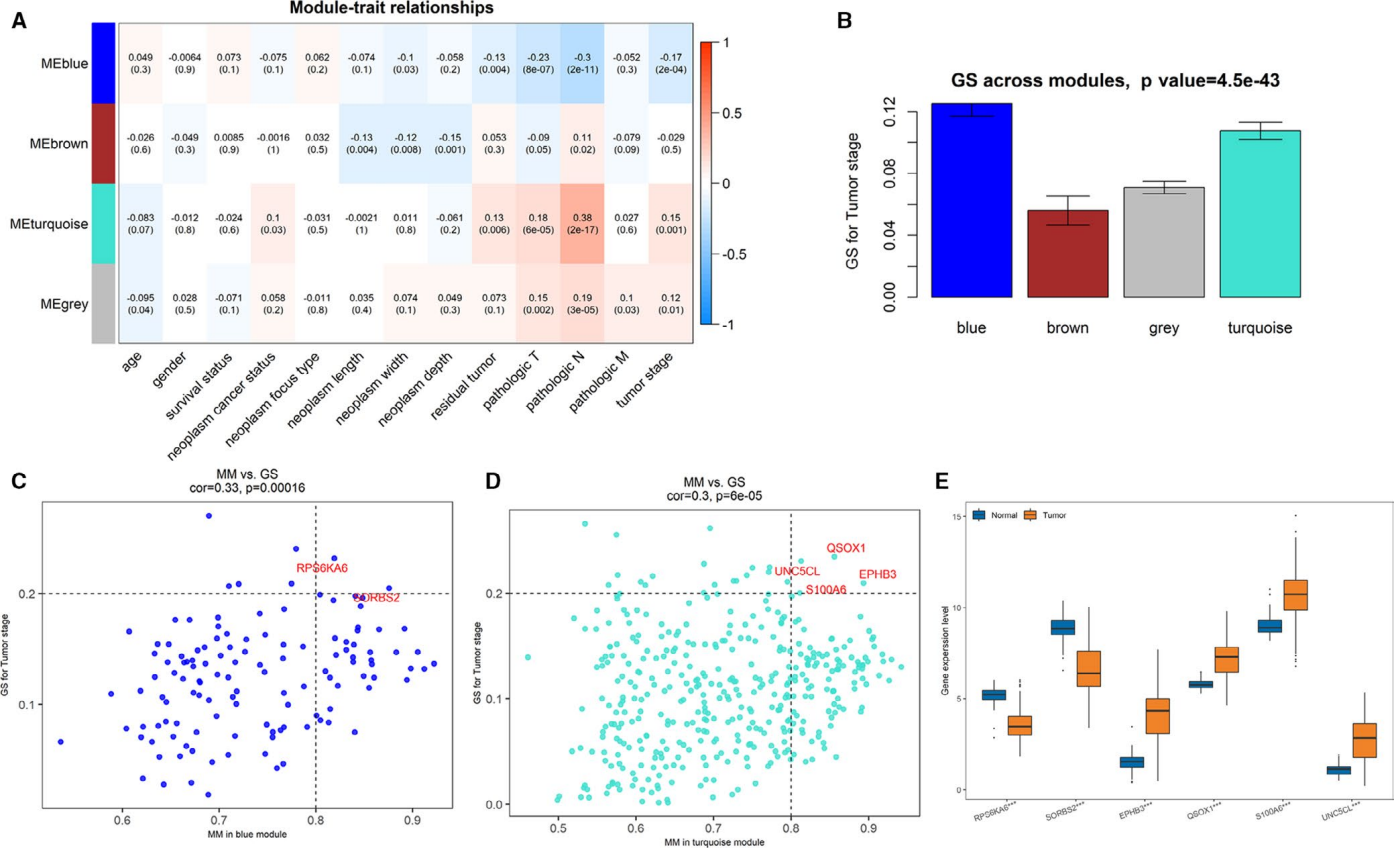
Identification of modules and hub genes associated with PTC tumour stage. (A) Heatmap of the correlation between module eigengenes and the clinical traits of PTC patients. (B) Correlation between gene modules and tumour stage. (C) Scatter plot of module eigengenes in blue module. (D) Scatter plot of module eigengenes in turquoise module. (The horizontal dashed line is at 0.2 and the vertical dashed line is at 0.8.; E) Expressions of 6 tumour stage‐related hub genes in PTC compared with normal tissues in the TCGA cohort (***: *p* < 0.001)

The module–clinical trait relationship analysis was conducted using ‘WGCNA’ package. In this study, 13 clinical traits of PTC patients contain age, gender, survival status, neoplasm cancer status, neoplasm focus type, neoplasm length, neoplasm width, neoplasm depth, residual tumour, pathologic T, pathologic *N*, pathologic M and tumour stage. As shown in Figure 3A, among these modules, brown module is correlated to neoplasm length (*r* ^2^ = −0.13, *p* = 0.004), neoplasm width (*r* ^2^ = −0.12, *p* = 0.008) and neoplasm depth (*r* ^2^ = −0.15, *p* = 0.001), while blue and turquoise modules show higher correlation with pathologic traits and tumour stage. Specifically, the blue module is related to pathologic T (*r* ^2^ = −0.23, *p* = 8e‐07), pathologic *N* (*r* ^2^ = −0.3, *p* = 2e‐11), and tumour stage (*r* ^2^ = −0.17, *p* = 2e‐04). The turquoise module is also correlated to pathologic T (*r* ^2^ = 0.18, *p* = 6e‐05), pathologic *N* (*r* ^2^ = 0.38, *p* = 2e‐17) and tumour stage (*r* ^2^ = 0.15, *p* = 0.001). Further, GS of each module for tumour stage also calculated. Figure 3B shows that GS values of blue and turquoise modules were much higher than brown module, so we can conclude that two modules (blue and turquoise) are confirmed to be associated with PTC pathological stages.

### 3.4 Validation of the hub genes

Based on criteria of GS >0.2 and MM >0.8, two genes (RPS6KA6 and SORBS2) in blue module and four genes (EPHB3, QSOX1, S100A6 and UNC5CL) in turquoise module were identified as hub genes (Figure 3C,D). Among them, RNA expressions of RPS6KA6 and SORBS2 in PTC tissues were significantly down‐regulated compared with normal tissues, while expressions of other four genes were significantly up‐regulated (Figure 3E). In order to verify this observation, expression levels of these 6 genes were also analysed based on three validation data sets of GEPIA database, GSE29265 and GSE3678, respectively (Figures S2‐S4). We can see that all six genes are differentially expressed in at least two data sets, especially RPS6KA6, SORBS2, EPHB3 and S100A6 in all data sets.

**FIGURE 4:**
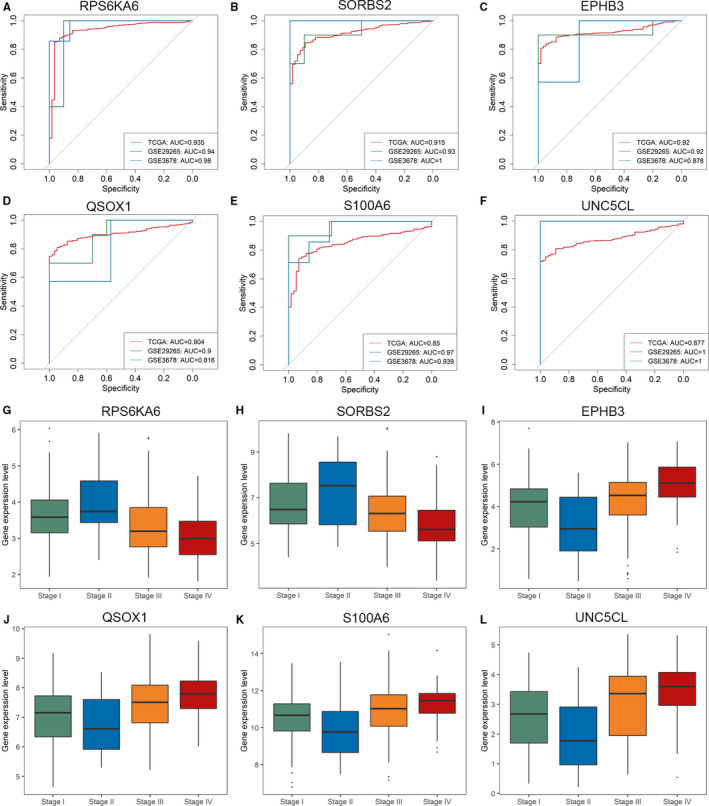
(A‐F) ROC curve analysis of 6 tumour stage‐related hub genes diagnosis in the TCGA, GSE29265 and GSE3678 cohort. (G‐L) Expressions of six tumour stage‐related hub genes in I, II, III and IV stages in the TCGA cohort

Recently, Park et al^21^ used penalized regression analysis and obtained an accurate model with 12 core pathway predictors for classifying PTC and normal thyroid tissues. When applied to the TCGA cohort, the model yielded an AUC values of 0.969. Likewise, the diagnostic performance of these six genes was also verified by ROC curve analysis. As shown in Figure 4A‐F, six AUC values are all higher than 0.85 and four over 0.90 in TCGA cohort. For two validation data sets of GSE29265 and GSE3678, almost all AUC values of the six hub genes are higher than 0.9, especially that of UNC5CL is equal to 1. These results illustrate that the six hub genes screened out by us also yield excellent diagnostic efficiency between PTC and normal tissues.

Besides, there are also significant differences on RNA expression levels of the six genes among four tumour stages (Figure 4G‐L), which were consistent with analysis results of the GEPIA database (Figure S5). Table 1 shows the *p* values of differential gene expression analysis between PTC stage I, stage II, stage III and stage IV, so the above validation tests suggest that the six hub genes are all reliable and potential biomarkers for marking different PTC stages. By deep literature‐exploring, all of six hub genes have been confirmed as important roles in cancers. The detailed function annotations are listed in Table 2.

**TABLE 1:** The *p* values among PTC stage I, stage II, stage III and stage IV by *T*‐test

Tumour stage	RPS6KA6	SORBS2	EPHB3	QSOX1	S100A6	UNC5CL
Stage I vs. Stage II	1.23E−02	1.53E−02	2.66E−04	4.37E−02	5.01E−04	1.67E−04
Stage I vs. Stage III	6.60E−03	4.77E−02	3.45E−02	1.92E−03	1.51E−03	6.06E−04
Stage I vs. Stage IV	2.17E−08	1.35E−07	1.56E−07	4.26E−07	9.34E−06	3.36E−07
Stage II vs. Stage III	4.45E−04	1.17E−03	2.41E−05	2.23E−04	1.29E−05	3.59E−07
Stage II vs. Stage IV	8.50E−09	1.04E−07	3.84E−10	2.78E−07	3.48E−08	6.68E−10
Stage III vs. Stage IV	7.35E−03	9.41E−04	2.05E−03	1.67E−02	1.60E−01	3.87E−02

**TABLE 2:** Detailed functional annotation about the six hub genes by deep literature‐exploring

Hub gene	Functional annotation
RPS6KA6	As a member of p90RSK family, it is closely associated with ERK, PI3K and p53 signalling pathways, as well as implicated in cell growth, survival, motility and senescence.^22^, ^23^, ^24^ It can mediate resistance to PI3K inhibitors in breast cancer cells both in vitro and vivo.^25^ It has been reported that RPS6KA6 is a prognostic factor for renal cell carcinoma (RCC) and its overexpression could promote cell cycle progression and enhance the invasive and metastatic capability of RCC cell lines^26^
SORBS2	SORBS2 (sorbin and SH3 domain containing 2) is an RNA binding protein. Previous studies have indicated that it is a tumour suppressor and can suppress the metastasis of many cancer. For example, it can suppresses metastatic colonization of ovarian cancer by stabilizing tumour‐suppressive immunomodulatory transcripts.^27^ Mediated by MEF2D, it suppresses the metastasis of human hepatocellular carcinoma by inhibiting the c‐Abl‐ERK signalling pathway,^28^ as well as hepatocellular carcinoma tumorigenesis^29^ and cervical carcinogenesis^30^
EPHB3	EPHB3 (Ephrin type‐B receptor 3) is one of EPH transmembrane tyrosine kinase receptors (TKRs) and has a critical function in tumour progression or regression in various cancers, such as colorectal cancer,^31^, ^32^, ^33^ non‐small‐cell lung cancer^34^, ^35^ and gastric cancer.^36^, ^37^, ^38^ In non‐small‐cell lung cancer, Li et al^34^ show that EPHB3 suppresses cancer cell metastasis via a PP2A/RACK1/Akt signalling complex. In contrast, Ji et al^35^ demonstrate that EPHB3 is overexpressed in this cancer and promotes tumour metastasis by enhancing cell survival and migration
QSOX1	QSOX1is an enzyme that oxidizes thiols during protein folding, reducing molecular oxygen to hydrogen peroxide, which may be utilized by tumour cells at different stages of tumorigenesis.^39^ The results of Sung et al^40^ have proven that QSOX1 might be a lung cancer tissue‐derived biomarker and be involved in the promotion of lung cancers, and thus can be a therapeutic target for lung cancers
S100A6	Overexpression of S100A6 is correlated with patient prognosis, so it is an independent prognostic predictor in gastric cancer and the methylation profile of specific CpG sites may affect its transcription.^41^ S100A6 can not only stimulate proliferation and migration of colorectal carcinoma cells through activation of the MAPK pathways,^42^ but also regulate the proliferation, invasion, migration and angiogenesis of lung cancer cells through the p53 acetylation.^43^ Moreover, it plays an important role in pancreatic cancer^44^, ^45^
UNC5CL	It is a novel inducer of a proinflammatory signalling cascade leading to activation of NF‐κB and JNK. It has been first described as a novel ZU5 and DD‐containing protein that is mostly homologous to the intracellular fragments of the Unc5‐receptor family members^46^

### 3.5 Genetic alteration analysis on hub genes

**FIGURE 5:**
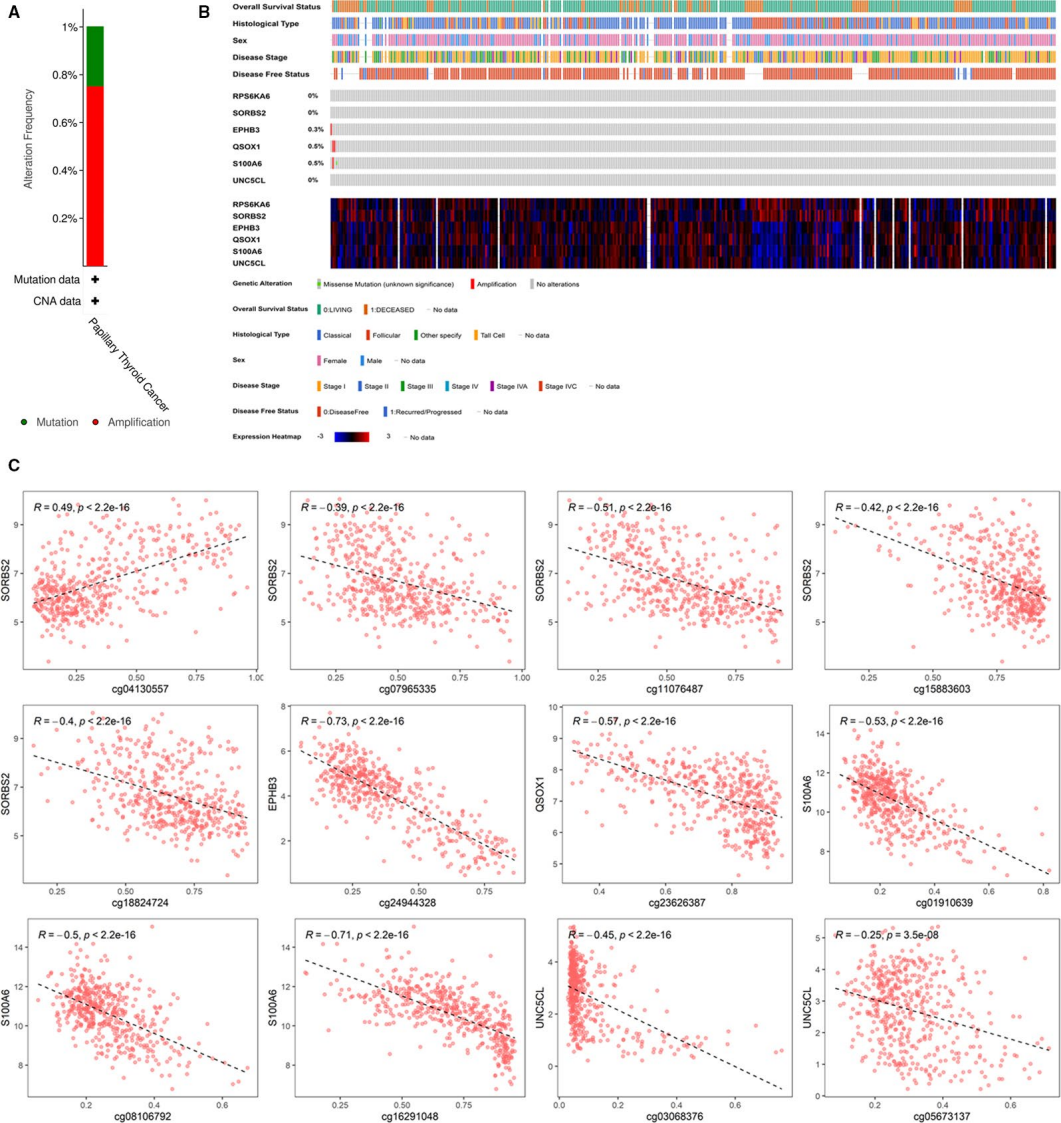
Genetic alterations associated with 6 tumour stage‐related hub genes. (A) Visual summary of Genetic alterations (data from PTC in TCGA) shows the genetic alteration of six hub genes. (B) The total alteration frequency of six hub genes. (C) Correlations between genes' expressions and DNA methylation values

We furtherly performed genetic alteration analysis for the six hub genes. The DNA mutations statuses of them were analysed using TCGA PTC patients' data in cBioPortal database. The six hub genes altered in about 4 (1%) of 399 PTC patients (Figure 5A), and the frequency of alteration of each gene is shown in Figure 5B. Only EPHB3, QSOX1 and S100A6 altered, but their frequencies of alteration were extremely low (0.3%, 0.5% and 0.5%, respectively; Figure 5B). These results indicated that mutations in the DNA of the six genes rarely occurred and they are all conservative.

Then, we studied the changes of DNA methylation sites on the six hub genes and their relationship with hub gene expressions. There are 195 DNA methylation sites on them. After data filtering and difference analysis, 16 DMSs were screened based on the criteria that |Δβ| > 0.1 and *p* value <0.05 (Table S1). Of them, 12 DMSs were found to be significantly associated with their corresponding genes' expression (Figure 5C and Table 3). The 12 DMSs could regulate their corresponding genes' expression levels. As shown in Figure 5C, only cg04130557 was positive correlation with expression level of its corresponding gene SORBS2, and others DMSs were all negative correlation with their corresponding genes' expression.

**TABLE 3:** Details of the differential methylation sites and corresponding genes

CpG_site	SiteLevel	GeneSymbol	GeneLevel	Relation	*R*	*p* value
cg24944328	Down	EPHB3	Up	Negative	−0.73	<2.2E−16
cg23626387	Down	QSOX1	Up	Negative	−0.57	<2.2E−16
cg01910639	Down	S100A6	Up	Negative	−0.53	<2.2E−16
cg08106792	Down	S100A6	Up	Negative	−0.5	<2.2E−16
cg16291048	Down	S100A6	Up	Negative	−0.71	<2.2E−16
cg04130557	Down	SORBS2	Down	Positive	0.49	<2.2E−16
cg07965335	Up	SORBS2	Down	Negative	−0.39	<2.2E−16
cg11076487	Up	SORBS2	Down	Negative	−0.51	<2.2E−16
cg15883603	Up	SORBS2	Down	Negative	−0.42	<2.2E−16
cg18824724	Up	SORBS2	Down	Negative	−0.4	<2.2E−16
cg03068376	Down	UNC5CL	Up	Negative	−0.45	<2.2E−16
cg05673137	Down	UNC5CL	Up	Negative	−0.25	3.50E−08

### 3.6 Construction of a prognostic signature for PTC stage III/IV patients

**FIGURE 6:**
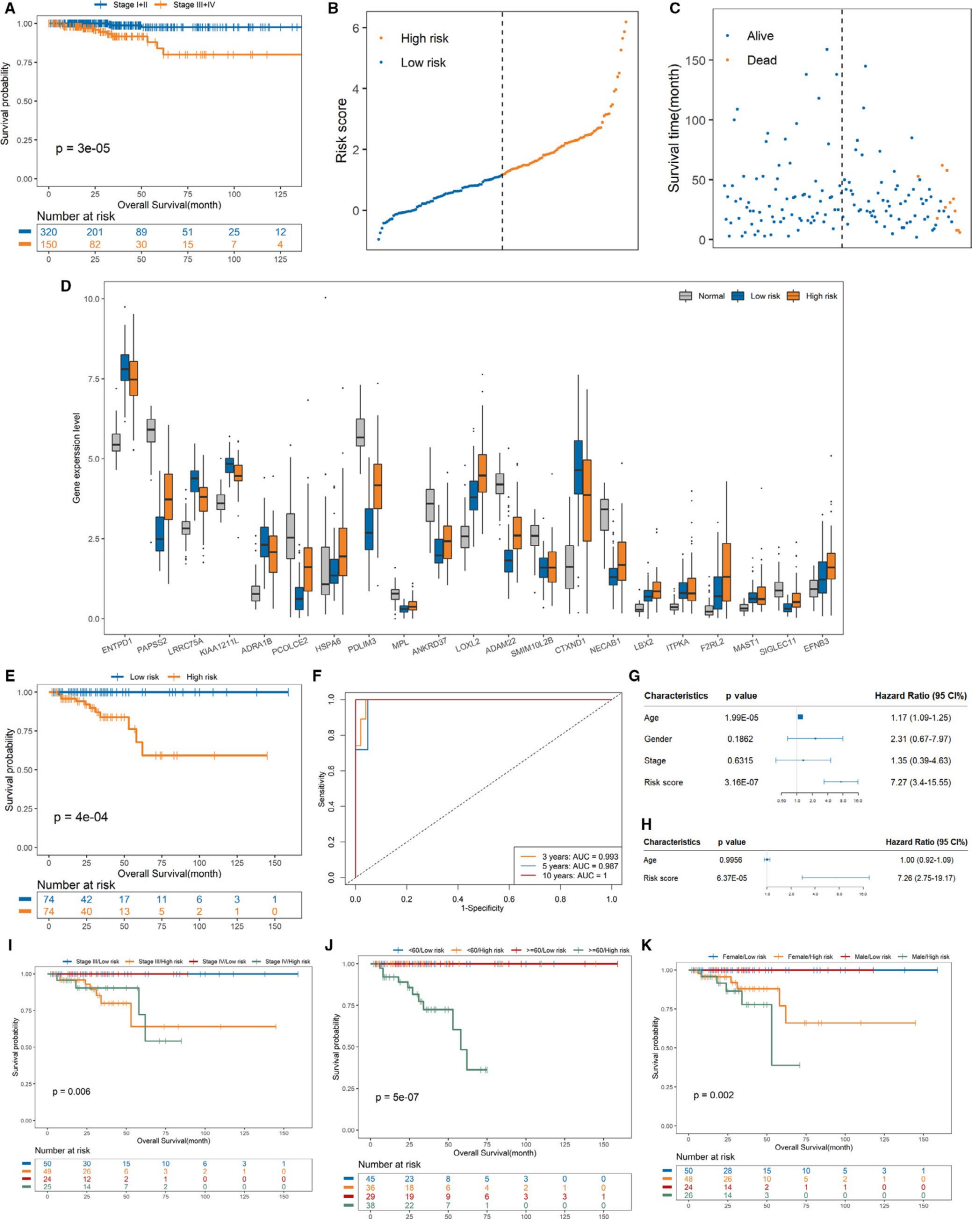
Construction and evaluation of the risk prognostic model for PTC advanced patients. (A) Kaplan‐Meier survival analysis of PTC patients between stage I+II and stage III+IV. (B) The distribution of the risk score. (C) The distribution of PTC advanced patients' follow‐up time and status. (D) Expressions distribution of the 21 genes in high‐risk, low‐risk and normal patients. (E) Kaplan‐Meier survival analysis of PTC advanced patients that are categorized into low‐risk and high‐risk groups using the median as the cut‐off. (F) The time‐dependent ROC curves of the risk score. (G) Forest plot summary of univariable analysis of age, gender, tumour stage and risk score. (H) Forest plot summary of multivariable analysis of age and risk score. (I) The Kaplan‐Meier curves for stage data set. (J) The Kaplan‐Meier curves for age data set. (K) The Kaplan‐Meier curves for gender data set

As shown in Figure 6A, we compared the survival of PTC patients with early stages (I and II) and those with advanced stages (III and IV), finding that the survival curve of early‐stage patients was significantly different from that of advanced patients and the survival time of advanced patients was significantly less than that of early patients. Therefore, it is of more significance to model the survival prognosis for PTC advanced patients. Initially, the six stage‐related genes were used to establish the prognosis model. But the univariate COX analysis results of six stage‐related hub genes show that the *p*‐values of six stage‐related genes are all much higher than 0.05, as listed in Table S2, so these genes give poor correlation with the survival prognosis of advanced PTC patients, which was further proved from Figure 6G that stage is not associated with PTC advanced patients' OS by univariate Cox regression analysis with *p*‐value of 0.6315.

Therefore, we extracted the expression data of the 1243 DEGs and survival information of 148 patients with PTC advanced stages. First, univariate Cox regression analysis was conducted. Results show that 230 genes were associated with PTC advanced patients' OS (*p* < 0.05). To further screen out an optimal combination from these genes, LASSO Cox regression analysis was performed and 21 genes were identified to develop a risk score model (Figure S6). Finally, using the coefficients derived from LASSO Cox algorithm, a risk score prognostic model was constructed based on RNA expression values of the 21 genes:

(2) $$\begin{array}{c} RiskScore=‐0.0990\times \text{ENTPD1}+0.0936\times \text{PAPSS2}‐0.3334\times \text{LRRC75A}‐0.1526\times \text{KIAA1211L}\\ ‐0.3278\times \text{ADRA1B}+0.2242\times \text{PCOLCE2}+0.1491\times \text{HSPA6}+0.0555\times \text{PDLIM3}+\\ 1.9123\times \text{MPL}+0.0247\times \text{ANKRD37}+0.0548\times \text{LOXL2}+0.0571\times \text{ADAM22}+\\ 0.2086\times \text{SMIM10L2B}‐0.0178\times \text{CTXND1}+0.2694\times \text{NECAB1}+1.2249\times \text{LBX2}+\\ 0.2555\times \text{ITPKA}+0.0851\times \text{F2RL2}+0.2510\times \text{MAST1}+0.8316\times \text{SIGLEC11}+\\ \;0.0701\times \text{EFNB3} \end{array}$$

The risk score of each patient was calculated, and all patients were divided into high‐ and low‐risk groups using the median as the cut‐off. The risk score profiles and survival time of each patient are shown in Figure 6B,C. We can observe all dead patients are in high‐risk group. In addition, the 21 gene expressions in normal, low‐risk and high‐risk group patients are shown in Figure 6D. It demonstrates that the expression levels of the 21 genes are all significantly different. From equation (2), the coefficients of 5 mRNAs are negative, so they are safety factors, while the coefficients of other 16 mRNAs are positive, so they are risk factors. For example, as shown in Figure 6D, the coefficient of ENTPD1 is negative and it is a safety factor, so the expression value in low‐risk patients is higher than that in high‐risk patients. While the coefficient of PAPSS2 is positive, which is a risk factor, the expression value in low‐risk patients is lower than that in high‐risk patients. The survival analysis indicated that high‐risk patients had shorter survival times than low‐risk patients (Figure 6E). In order to further assess the performance of this prognosis model on the survival time prediction of PTC advanced patients, we conducted time‐dependent ROC analysis of 3‐, 5‐ and 10‐year (Figure 6F). The three AUC values were 0.993, 0.987 and 1 at 3, 5 and 10 years, respectively, suggesting that the model constructed based on the 21 genes yields the strong prognostic ability.

Consequently, we aim to confirm that the prognostic signature is of high applicability and could precisely predict the OS of PTC‐advanced patients. As shown in Figure 6G, univariate Cox regression analysis reveals that both age and risk score are associated with TPC‐advanced patients' OS, but multivariate Cox regression analysis show that the risk score is an independent prognostic predictor for OS with HR, 95% CI and *p*‐value of 7.26, 2.75–19.17 and 6.37 × 10^−5^, respectively (Figure 6H).

Then, the stratification analysis was implemented based on age, gender and tumour stage. The patients were divided into four subgroups based on stage III/low‐risk, stage III/high‐risk, stage IV/low‐risk and stage IV/high‐risk, as shown in Figure 6I. The result indicates both stage III and IV patients in high‐risk group have poorer OS than low‐risk patients. Meanwhile, based on age and gender, patients were divided into four subgroups (<60/low‐risk, <60/high‐risk, ≥60/low‐risk and ≥60/high‐risk) and four subgroups (female/low‐risk, female/high‐risk, male/low‐risk and male/high‐risk). As expected, OS of ≥60/high‐risk group patients is the worst (Figure 6J). In addition, in both female and male groups, high‐risk patients have shorter survival time than low‐risk ones (Figure 6K). Overall, this prognostic signature shows a satisfactory applicability when advanced patients are regrouped by different clinicopathological characteristic, suggesting that it is an independent applicable prognostic predictor for PTC‐advanced patients.

### 3.7 Biological pathway and tumour microenvironment alteration between high‐ and low‐risk patients

**FIGURE 7:**
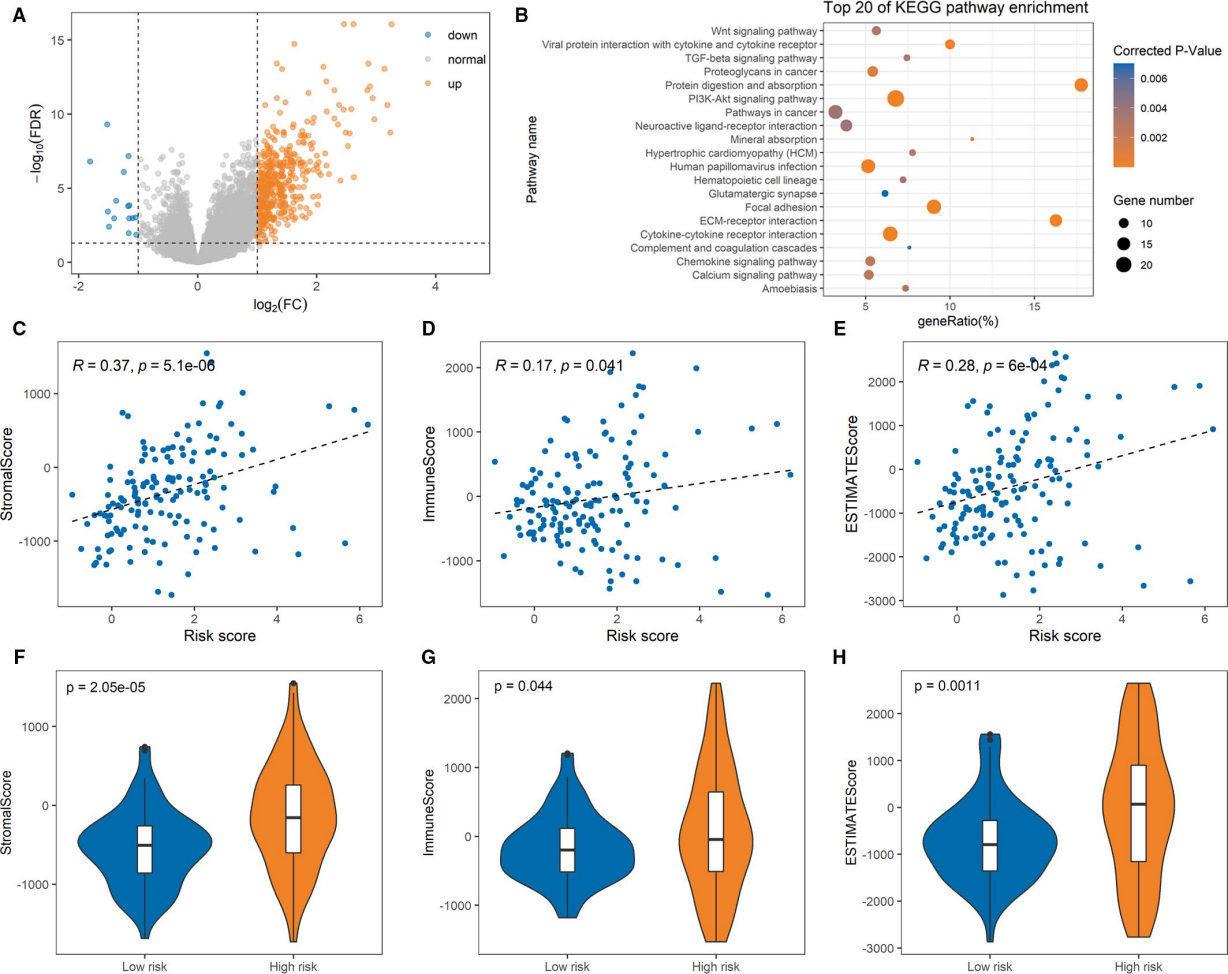
Kyoto Encyclopedia of Genes and Genomes (KEGG) pathway and tumour microenvironment analyses between high‐risk and low‐risk patients. (A) Volcano plot of differentially expressed genes between high‐risk and low‐risk patients. (B) The top 20 KEGG pathways enriched of differentially expressed genes. (C‐E) Correlations between the risk score and Stromal Score, Immune Score and ESTIMATE Score in the TCGA cohort, respectively. (F‐H) Comparison of the Stromal Score, Immune Score and ESTIMATE Score between low‐risk and high‐risk patients in the TCGA cohort, respectively

To explore potential biological pathway alteration between high‐ and low‐risk patients, we conducted KEGG pathway enrichment analysis on DEGs between two groups. First, according to |log_2_FC| ≥ 1 and FDR<0.05, we obtained 454 DEGs, including 439 up‐regulated and 15 down‐regulated genes, as shown in Figure 7A. Here, 40 pathways were enriched on these 454 DEGs, as listed in Table S3. Figure 7B shows the top 20 enriched pathways. We can see that pathways associated with cancers were enriched, such as ‘Wnt signalling pathway’, ‘TGF‐beta signalling pathway’, ‘Proteoglycans in cancer’, ‘PI3K‐Akt signalling pathway’ and ‘Pathways in cancer’, illustrating that these DEGs may promote the progress of PTC to some extent.

As we know, understanding the tumour microenvironment (TME) is of practical significance for cancer diagnosis and treatments. As major fraction of TME, infiltrating stromal and immune cells form the major non‐tumour constituents of tumour tissues, which not only perturb the tumour signal in molecular studies but also have an important role in cancer biology.^20^ Therefore, in order to investigate the relationship between these cells and the prognostic signature, ESTIMATE was preformed to calculated the Stromal Score, Immune Score and ESTIMATE Score for 148 PTC‐advanced patients using R package ‘estimate’. Here, the higher value estimated in Immune Score or Stromal Score means to the larger amount of the immune or stromal components in TME. ESTIMATE Score is the sum of Immune Score and Stromal Score denoting the comprehensive proportion of both components in TME. First, we analysed whether these scores were correlated with the risk score. As shown in Figure 7C–E, the prognostic signature is significantly positively correlated with Stromal Score, Immune Score and ESTIMATE Score (*p* < 0.05) respectively. Then, we performed difference analysis in terms of Stromal, Immune and ESTIMATE scores between low‐ and high‐risk patients. Figure 7F‐H demonstrate that high‐risk patients have higher Stromal, Immune and ESTIMATE scores (*p* < 0.05). These results suggest that TME of high‐risk patients, compared with low‐risk patients, may be more conducive to tumour growth.

## 4 DISCUSSION

In this paper, we systematically analysed PTC tumour stage‐related genes and constructed a prognostic risk signature for PTC stage III/IV patients. The workflow of this study is shown in Figure S7. Based on 1243 DEGs, three co‐expression gene modules were achieved by WGCNA analysis. GO and KEGG pathway enrichment analysis were performed on the three modules, which indicates that they are all related with cancer and immune pathways. Of them, two were identified to be closely related to pathologic stages by module–clinical trait relationship analysis. The genes in both two modules were mainly enriched in cancer‐related pathways, such as ‘PI3K‐Akt signalling pathway’, ‘MAPK signalling pathway’ and ‘Jak‐STAT signalling pathway’. These results illustrate that although genes in two modules have different gene expression patterns, they are similar in biological pathways and play a similar role in the development of PTC.

Six hub genes of RPS6KA6, SORBS2, EPHB3, QSOX1, S100A6 and UNC5CL from the two stage‐related modules were identified and then underwent comprehensive validation tests, including expression difference analysis between tumour and normal tissue in our data set, GEPIA database, GSE29265 and GSE3678, as well as among four stage tumours based on our data set and GEPIA database, respectively. Moreover, ROC curve analysis shows that these six hub genes yield excellent diagnostic efficiency between tumour and normal tissues. The alteration statuses of six hub genes were also analysed and mutations in the DNA of the six genes rarely occur, indicating that they are all much conservative, but the changes of DMSs on the six genes show that 12 DMSs are significantly associated with their corresponding genes' expression, so DNA methylation on six genes should be paid close attention in following researches. Finally, by deep literature‐exploring as described in Table 2, all of the six hub genes have been confirmed as important roles in cancers. All above analysis prove that these six hub genes would be potential biomarkers for PTC diagnosis and marking PTC stages.

Tang et al.^7^ have also given five hub genes for PTC (COL1A1, COL1A2, COL3A1, COL5A2 and DCN) by WGCNA and protein‐protein interaction network methods, but further validation about them needs to be explored. Since we performed more rigorous data filtering, COL1A2 is absent in our data set. Here, we conducted ROC curve analysis and expression difference analysis on other four genes. Figure S8 shows that AUC values of four genes are 0.757, 0.642, 0.696 and 0.909, respectively, while those of our six hub genes are all greater than 0.85 and four higher than 0.9 (Figure 4A‐F). The expression levels of the four genes yield no significant difference (Figure S9 and Table S4), compared with those of the six genes listed in Table 1. Based on different data filtering, there is no overlap of hub genes between our work and Tang et al., so the practical applicability of those genes would be experimentally confirmed in the future researches.

Survival curve reveals that the survival time of patients with advanced stage (III and IV) is significantly less than that of patients with early stage (I and II). Therefore, prognostic risk modelling for patients with advanced stage is more important for precise medical treatments. From common 1243 DEGs, 230 genes are associated with PTC‐advanced patients' OS by univariate regression analysis. Among them, 21 genes were identified to develop a risk score model by LASSO Cox algorithm. This prognostic signature can successfully divide PTC patients into high‐ and low‐risk groups and is independent of the clinical indicators by stratification analysis. Consequently, biological pathway alteration analysis on DGEs between high‐ and low‐risk groups illustrate that these DEGs promote the progress of PTC to some extent. Meanwhile, high‐risk patients have higher stromal, immune and ESTIMATE scores than low‐risk patients, suggesting that TME of high‐risk patients may be more conducive to tumour growth. We can conclude the 21‐mRNA‐based prognostic risk signature could be a novel and effectively independent prognosis signature for predicting survival in advanced patients with PTC.

## CONFLICT OF INTEREST

The authors confirm that there are no conflicts of interest.

## AUTHOR CONTRIBUTIONS

**Lei Xu:** Data curation (equal); Formal analysis (equal); Methodology (equal); Software (equal); Validation (equal); Writing‐original draft (equal). **Feng Liu:** Data curation (equal); Investigation (equal); Methodology (equal); Resources (equal); Supervision (equal). **Haiyan Li:** Data curation (equal); Methodology (equal); Resources (equal). **Menglong Li:** Methodology (equal); Resources (equal); Validation (equal). **Yongmei Xie:** Data curation (equal); Methodology (equal); Resources (equal). **Zhihui Li:** Conceptualization (equal); Funding acquisition (equal); Supervision (equal); Writing‐review & editing (equal). **Yanzhi Guo:** Conceptualization (equal); Formal analysis (equal); Investigation (equal); Supervision (equal); Writing‐original draft (equal); Writing‐review & editing (equal).

## Supporting information

Supplementary Material

## Data Availability

Data sharing is not applicable to this article as no new data were created or analysed in this study.
