# Supplementary material for: Comprehensive characterization of pathological stage‐related genes of papillary thyroid cancer along with survival prediction
Source: J Cell Mol Med. 2021 Aug 2;25(17):8390–404. doi: 10.1111/jcmm.16799 (PMC8419169; doi:10.1111/jcmm.16799)
Supplement: Supplementary file 1 — Supplementary Material [file JCMM-25-8390-s001.doc]

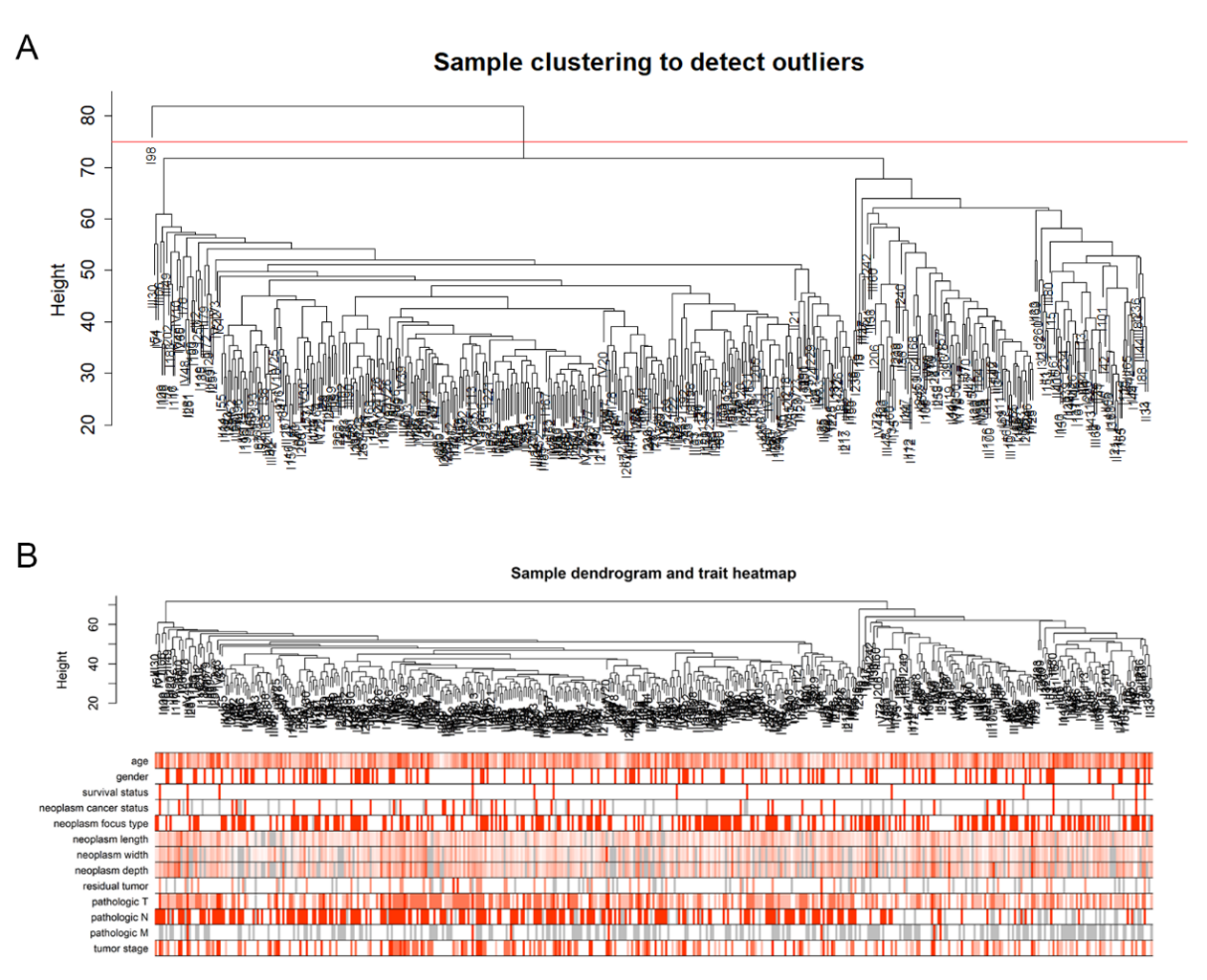


**Supplementary Figure 1.** Clustering dendrogram of PTC tumor samples and the clinical traits


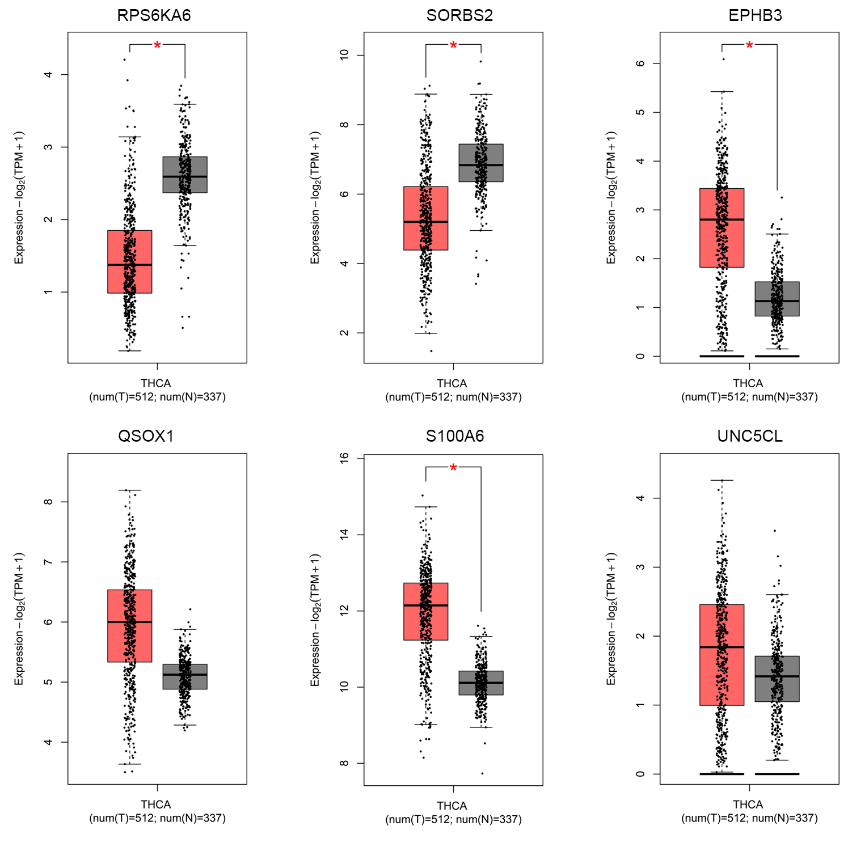


**Supplementary Figure 2.** Expressions of 6 tumor stage-related hub genes in PTC compared with normal tissues in the GEPIA database. (*: (|log_2_FC|) ≥ 1 and p < 0.05)


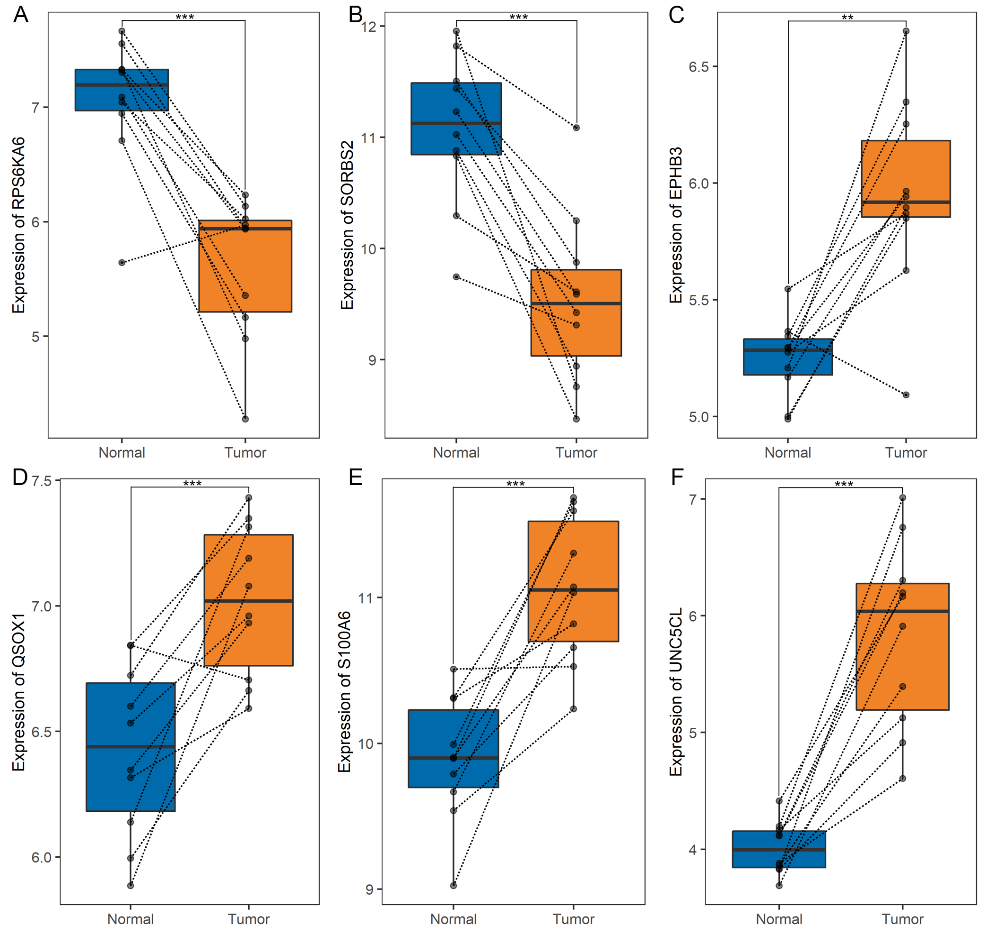


**Supplementary Figure 3.** Expressions of 6 tumor stage-related hub genes in PTC compared with normal tissues in the GSE29265 dataset. (*: p < 0.05, **: p < 0.01, ***: p < 0.001)


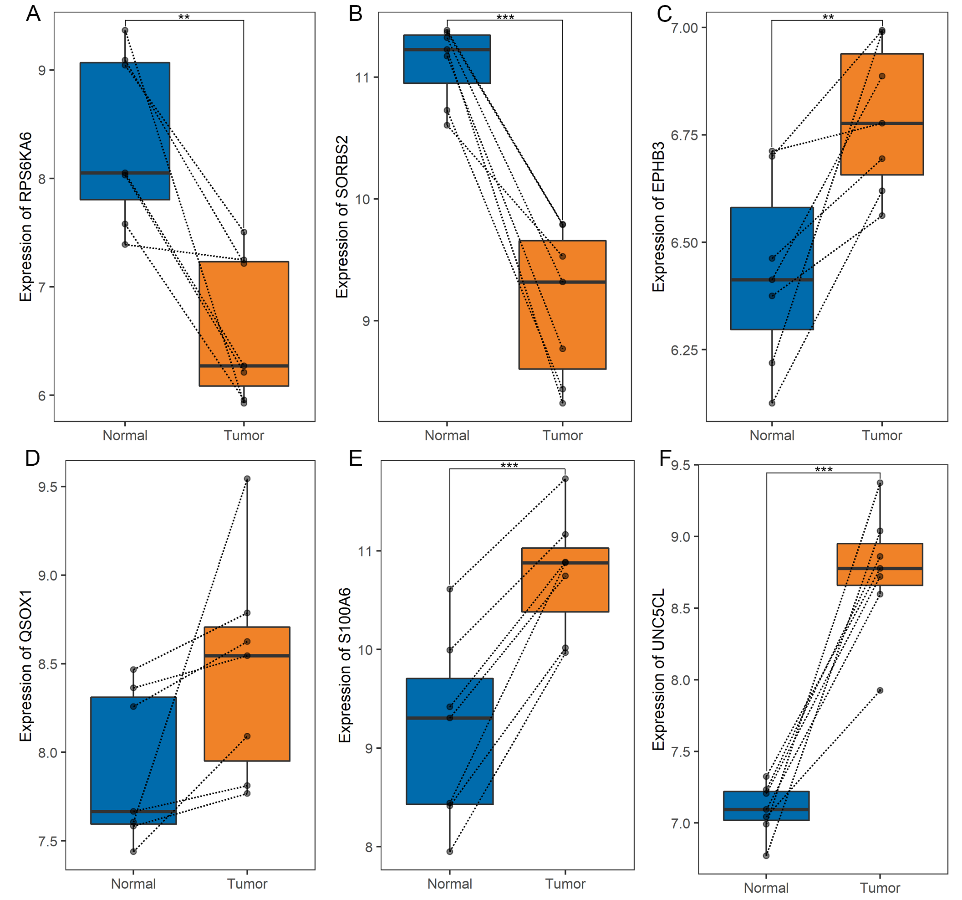


**Supplementary Figure 4.** Expressions of 6 tumor stage-related hub genes in PTC compared with normal tissues in the GSE3678 dataset. (*: p < 0.05, **: p < 0.01, ***: p < 0.001)


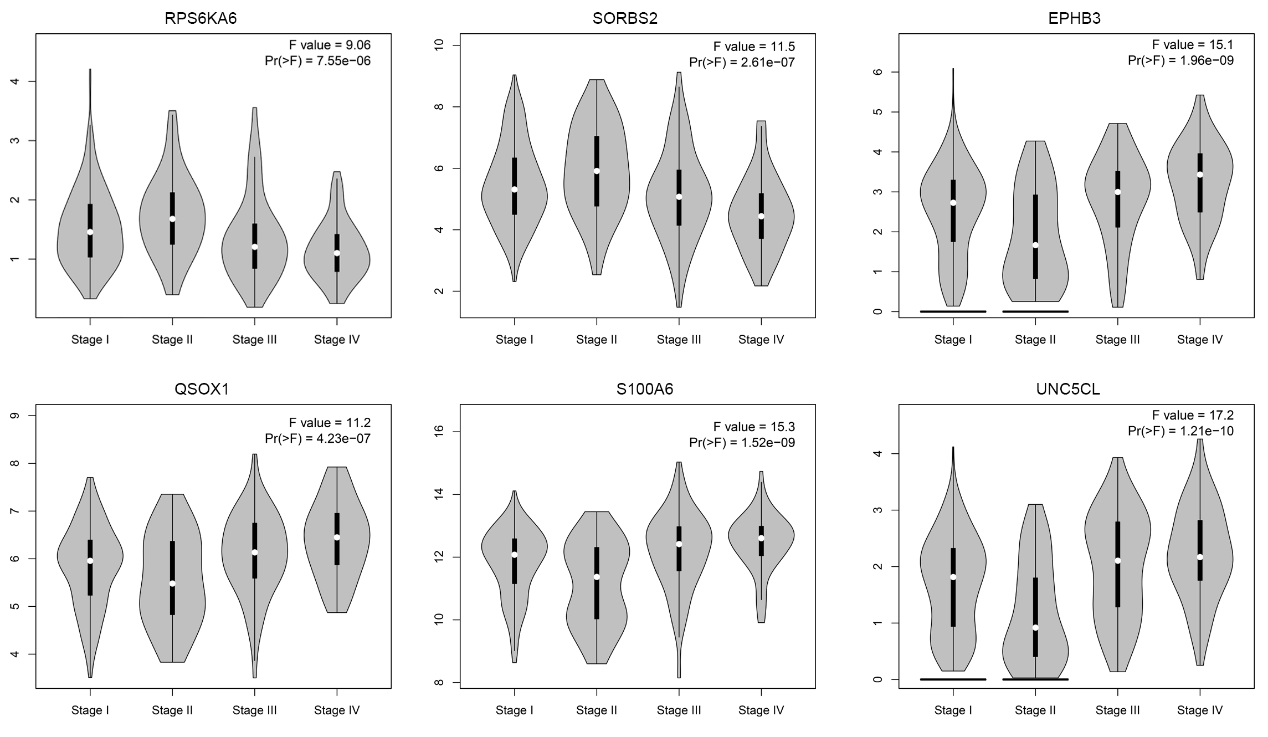


**Supplementary Figure 5.** Expressions of 6 tumor stage-related hub genes in I, II, III and IV stages in the GEPIA database.


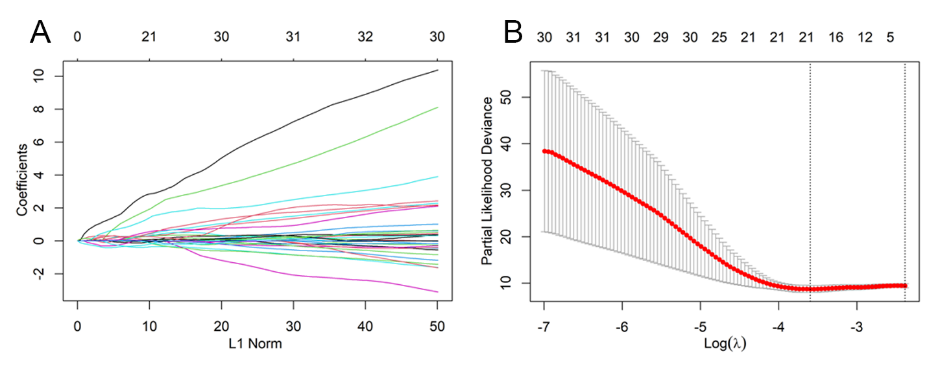


**Supplementary Figure 6.** Construction the risk prognostic model for PTC advanced patients. (A) The screening process for 21 genes using LASSO Cox method. (B) The plot of the partial likelihood deviance versus log λ, where λ is the tuning parameter (Here, the optimal λ value is 0.0217).


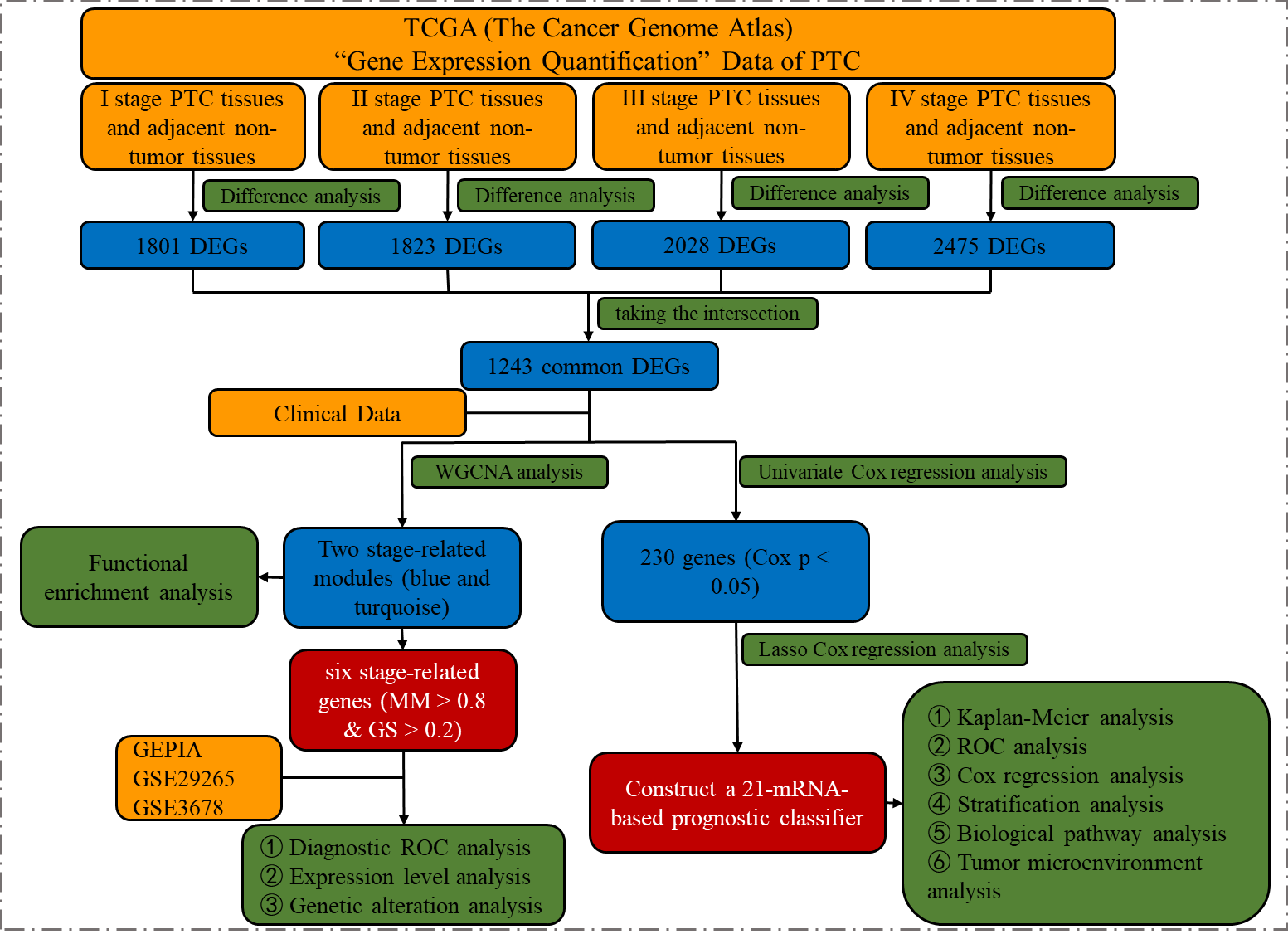


**Supplementary Figure 7.** Overview of the analysis procedure, including collection, preprocessing, analysis and validation.


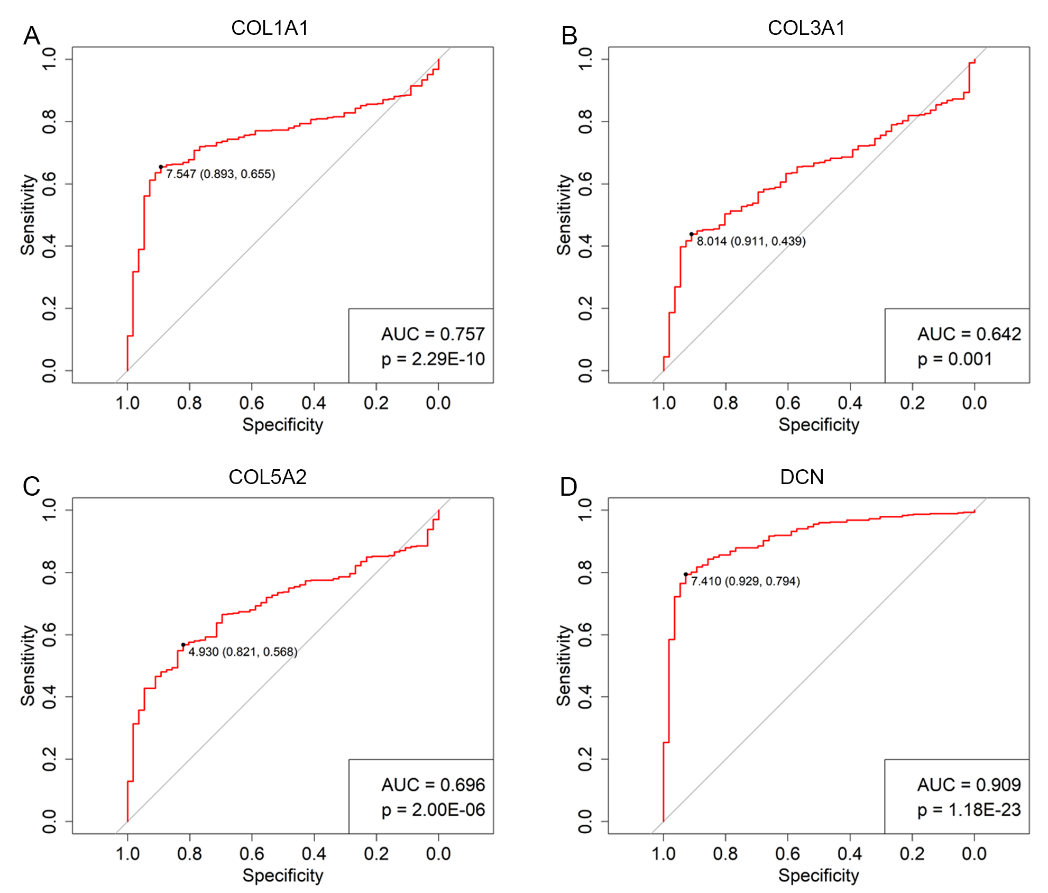


**Supplementary Figure 8.** (A-D) ROC curve analysis of COL1A1, COL3A1, COL5A2 and DCN diagnosis.


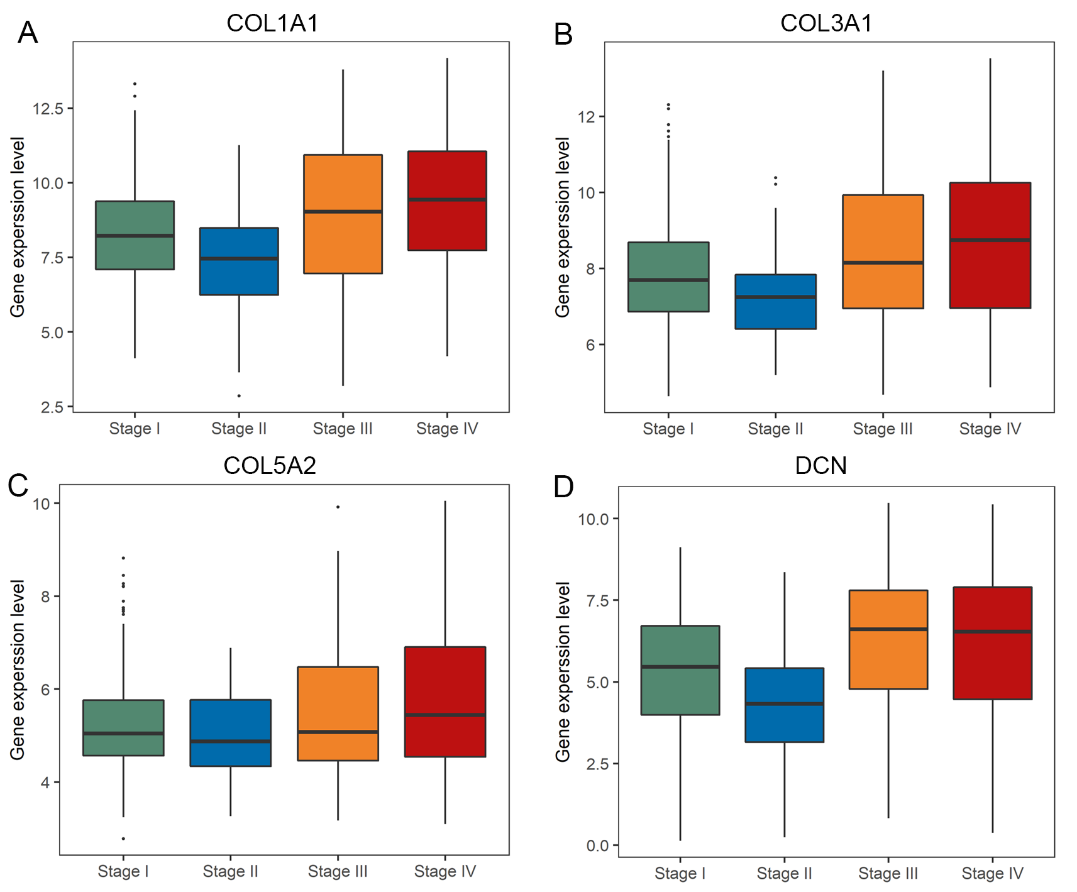


**Supplementary Figure 9.** (A-D) Expressions of COL1A1, COL3A1, COL5A2 and DCN in I, II, III and IV stages in the TCGA cohort.

**Supplementary Table S1.** Details of the differential methylation sites

| No | CpG_site | Δβ | p value | Gene_Symbol | Chromosome | Gene_Type | Feature_Type | Start | End |
| --- | --- | --- | --- | --- | --- | --- | --- | --- | --- |
| 1 | cg24944328 | -0.21 | 7.43E-15 | EPHB3 | chr3 | protein_coding | S_Shelf | 184565520 | 184565521 |
| 2 | cg23626387 | -0.11 | 2.50E-07 | QSOX1 | chr1 | protein_coding | . | 180167752 | 180167753 |
| 3 | cg01910639 | -0.13 | 7.91E-16 | S100A6 | chr1 | protein_coding | N_Shore | 153535303 | 153535304 |
| 4 | cg08106792 | -0.11 | 3.12E-15 | S100A6 | chr1 | protein_coding | S_Shore | 153536847 | 153536848 |
| 5 | cg16291048 | -0.15 | 1.11E-08 | S100A6 | chr1 | protein_coding | N_Shore | 153535022 | 153535023 |
| 6 | cg04130557 | -0.15 | 1.87E-06 | SORBS2 | chr4 | protein_coding | . | 185839224 | 185839225 |
| 7 | cg07965335 | 0.14 | 5.03E-08 | SORBS2 | chr4 | protein_coding | . | 185788107 | 185788108 |
| 8 | cg11076487 | 0.18 | 8.21E-12 | SORBS2 | chr4 | protein_coding | . | 185788084 | 185788085 |
| 9 | cg15883603 | 0.27 | 1.76E-38 | SORBS2 | chr4 | protein_coding | . | 185701254 | 185701255 |
| 10 | cg18824724 | 0.15 | 6.22E-12 | SORBS2 | chr4 | protein_coding | . | 185776580 | 185776581 |
| 11 | cg03068376 | -0.11 | 1.03E-11 | UNC5CL | chr6 | protein_coding | N_Shore | 41027904 | 41027905 |
| 12 | cg05673137 | -0.17 | 1.46E-25 | UNC5CL | chr6 | protein_coding | . | 41039264 | 41039265 |
| 13 | cg27257822 | 0.11 | 8.73E-06 | EPHB3 | chr3 | protein_coding | S_Shore | 184575092 | 184575093 |
| 14 | cg00028336 | 0.18 | 1.17E-14 | SORBS2 | chr4 | protein_coding | . | 185820634 | 185820635 |
| 15 | cg11956467 | 0.17 | 3.84E-21 | SORBS2 | chr4 | protein_coding | . | 185820985 | 185820986 |
| 16 | cg16433265 | 0.12 | 8.62E-08 | SORBS2 | chr4 | protein_coding | . | 185820661 | 185820662 |

**Supplementary Table S2.** Univariate COX regression analysis results of 6 stage-related hub genes.

| Gene | Coefficient | Hazard Ratio | 95% CI | P Value |
| --- | --- | --- | --- | --- |
| RPS6KA6 | -0.1098 | 0.9 | 0.42-1.93 | 0.778813 |
| SORBS2 | 0.2034 | 1.23 | 0.78-1.92 | 0.376513 |
| EPHB3 | -0.1183 | 0.89 | 0.6-1.31 | 0.553322 |
| QSOX1 | 0.0198 | 1.02 | 0.5-2.1 | 0.957252 |
| S100A6 | -0.226 | 0.8 | 0.53-1.21 | 0.285091 |
| UNC5CL | 0.0168 | 1.02 | 0.6-1.73 | 0.950855 |

**Supplementary Table S3.** The KEGG pathways of differentially expressed genes between high- and low-risk patients.

| Pathway name | ID | Input number | Background number | Gene Ratio(%) | P value | Corrected P value |
| --- | --- | --- | --- | --- | --- | --- |
| Protein digestion and absorption | hsa04974 | 16 | 90 | 17.77778 | 8.86E-14 | 1.45E-11 |
| ECM-receptor interaction | hsa04512 | 14 | 86 | 16.27907 | 9.14E-12 | 1.22E-09 |
| PI3K-Akt signaling pathway | hsa04151 | 24 | 354 | 6.779661 | 1.69E-11 | 2.16E-09 |
| Focal adhesion | hsa04510 | 18 | 199 | 9.045226 | 8.00E-11 | 8.73E-09 |
| Cytokine-cytokine receptor interaction | hsa04060 | 19 | 294 | 6.462585 | 4.63E-09 | 4.13E-07 |
| Viral protein interaction with cytokine and cytokine receptor | hsa04061 | 10 | 100 | 10 | 5.64E-07 | 2.97E-05 |
| Human papillomavirus infection | hsa05165 | 17 | 330 | 5.151515 | 6.32E-07 | 3.21E-05 |
| Proteoglycans in cancer | hsa05205 | 11 | 203 | 5.418719 | 3.89E-05 | 0.001091 |
| Mineral absorption | hsa04978 | 6 | 53 | 11.32075 | 5.70E-05 | 0.001435 |
| Chemokine signaling pathway | hsa04062 | 10 | 190 | 5.263158 | 0.000109 | 0.002452 |
| Calcium signaling pathway | hsa04020 | 10 | 193 | 5.181347 | 0.000123 | 0.002709 |
| Hypertrophic cardiomyopathy (HCM) | hsa05410 | 7 | 90 | 7.777778 | 0.000126 | 0.002748 |
| Wnt signaling pathway | hsa04310 | 9 | 160 | 5.625 | 0.000149 | 0.003135 |
| TGF-beta signaling pathway | hsa04350 | 7 | 94 | 7.446809 | 0.000162 | 0.003275 |
| Amoebiasis | hsa05146 | 7 | 95 | 7.368421 | 0.000173 | 0.003316 |
| Hematopoietic cell lineage | hsa04640 | 7 | 97 | 7.216495 | 0.000195 | 0.003538 |
| Pathways in cancer | hsa05200 | 17 | 530 | 3.207547 | 0.000213 | 0.00382 |
| Neuroactive ligand-receptor interaction | hsa04080 | 13 | 338 | 3.846154 | 0.000224 | 0.003868 |
| Complement and coagulation cascades | hsa04610 | 6 | 79 | 7.594937 | 0.000432 | 0.006418 |
| Glutamatergic synapse | hsa04724 | 7 | 114 | 6.140351 | 0.000491 | 0.007012 |
| Phagosome | hsa04145 | 8 | 152 | 5.263158 | 0.000526 | 0.007365 |
| Dilated cardiomyopathy (DCM) | hsa05414 | 6 | 96 | 6.25 | 0.001131 | 0.012513 |
| Primary immunodeficiency | hsa05340 | 4 | 37 | 10.81081 | 0.001204 | 0.01317 |
| Pancreatic secretion | hsa04972 | 6 | 98 | 6.122449 | 0.001251 | 0.013481 |
| AGE-RAGE signaling pathway in diabetic complications | hsa04933 | 6 | 100 | 6 | 0.001379 | 0.014656 |
| Hippo signaling pathway | hsa04390 | 7 | 154 | 4.545455 | 0.002575 | 0.022912 |
| Malaria | hsa05144 | 4 | 49 | 8.163265 | 0.003127 | 0.026541 |
| Cocaine addiction | hsa05030 | 4 | 49 | 8.163265 | 0.003127 | 0.026541 |
| Jak-STAT signaling pathway | hsa04630 | 7 | 162 | 4.320988 | 0.00337 | 0.026809 |
| Platelet activation | hsa04611 | 6 | 124 | 4.83871 | 0.003843 | 0.028779 |
| Relaxin signaling pathway | hsa04926 | 6 | 130 | 4.615385 | 0.004786 | 0.033064 |
| Rheumatoid arthritis | hsa05323 | 5 | 91 | 5.494505 | 0.004903 | 0.033557 |
| IL-17 signaling pathway | hsa04657 | 5 | 93 | 5.376344 | 0.005348 | 0.035367 |
| Metabolic pathways | hsa01100 | 28 | 1433 | 1.953943 | 0.005552 | 0.035679 |
| Nicotinate and nicotinamide metabolism | hsa00760 | 3 | 30 | 10 | 0.00621 | 0.038803 |
| C-type lectin receptor signaling pathway | hsa04625 | 5 | 104 | 4.807692 | 0.008318 | 0.046537 |
| Toll-like receptor signaling pathway | hsa04620 | 5 | 104 | 4.807692 | 0.008318 | 0.046537 |
| Phospholipase D signaling pathway | hsa04072 | 6 | 148 | 4.054054 | 0.008633 | 0.047937 |
| Retinol metabolism | hsa00830 | 4 | 67 | 5.970149 | 0.008845 | 0.04884 |
| Amphetamine addiction | hsa05031 | 4 | 68 | 5.882353 | 0.009283 | 0.049601 |

**Supplementary Table S4.** The p values among PTC stage I, stage II, stage III and stage IV by T-test.

| Tumor stage | COL1A1 | COL3A1 | COL5A2 | DCN |
| --- | --- | --- | --- | --- |
| Stage I vs. Stage II | 9.68E-04 | 6.59E-03 | 2.08E-01 | 4.02E-04 |
| Stage I vs. Stage III | 4.68E-03 | 2.37E-02 | 2.11E-02 | 1.57E-03 |
| Stage I vs. Stage IV | 2.69E-05 | 4.19E-04 | 5.42E-04 | 5.04E-02 |
| Stage II vs. Stage III | 3.50E-05 | 8.07E-04 | 2.14E-02 | 4.99E-07 |
| Stage II vs. Stage IV | 1.86E-06 | 1.31E-04 | 3.83E-03 | 2.33E-04 |
| Stage III vs. Stage IV | 1.32E-01 | 1.97E-01 | 2.27E-01 | 7.57E-01 |
